# Comparison of Side Effects Between 3‐Monthly and 1‐Monthly Paliperidone Palmitate Formulations in Patients With Schizophrenia

**DOI:** 10.1002/hup.70033

**Published:** 2026-01-08

**Authors:** Erkan Kuru, Ilker Ozdemir, Bengu Yucens, M. Hakan Türkçapar

**Affiliations:** ^1^ Private Practice, Psychiatry Ankara Turkey; ^2^ Department of Psychiatry Bursa City Hospital Bursa Turkey; ^3^ Department of Psychiatry Faculty of Medicine Pamukkale University Denizli Turkey; ^4^ Department of Psychology Social Sciences University of Ankara Ankara Turkey

**Keywords:** antipsychotic agents, drug‐related side effects and adverse reactions, long‐acting injectable, paliperidone palmitate 3‐month, psychopharmacology, schizophrenia, treatment outcome

## Abstract

**Background:**

The paliperidone palmitate 3‐month (PP3M) formulation offers an extended dosing interval compared to the 1‐month (PP1M) formulation. However, data on the comparative side effect profiles of PP1M versus PP3M in real‐world settings remain limited. This study aimed to compare the side effect profiles in patients with schizophrenia receiving PP1M and those who switched from PP1M to PP3M.

**Material and Methods:**

Of 473 patients with schizophrenia screened, 132 received long‐acting injectable antipsychotics; 67 initiated PP1M, of whom 43 subsequently converted to PP3M and had evaluable data at both time points. The primary analysis used a within‐patient mirror‐image design (PP1M month‐4 vs PP3M month‐4). Side effects were assessed using the UKU Side Effect Rating Scale, and symptom severity was evaluated with the Scale for the Assessment of Negative Symptoms and the Scale for the Assessment of Positive Symptoms, at 4 months after PP1M initiation and 4 months after PP3M switch.

**Results:**

UKU side‐effect profiles were broadly similar after conversion to PP3M, with no increase in the UKU total score. The most frequent side effects for both formulations were increased fatigability, hypokinesia, weight gain, and diminished sexual desire. The frequency of side effects remained mostly stable, with constipation showing a significant decrease after switching. Side effects were more prevalent in patients with schizophrenia using multiple antipsychotics compared to monotherapy.

**Conclusion:**

In this within‐patient cohort stabilized on PP1M, conversion to PP3M was not associated with an increase in overall UKU side‐effect burden. Selecting the appropriate dose before switching and preferring monotherapy may enhance treatment comfort. These results may help guide clinicians in selecting long‐acting injectable antipsychotics in practice.

## 1 Introduction

Effective management of schizophrenia typically involves consistent administration of antipsychotic medications: among individuals experiencing a first episode of schizophrenia, the risk of relapse reaches approximately 70% in those who do not comply with their medication regimen, whereas this rate drops to around 25% in patients who maintain adherence to their prescribed treatment (Patel and David 2005). Despite this, nearly 50% of patients discontinue oral antipsychotic (OAP) therapy within the first year post‐discharge, and the proportion of those continuing treatment falls below 30% in the second year (Waddell and Taylor 2009; Zygmunt et al. 2002). In this context, long‐acting injectable antipsychotics (LAIAs) have been shown to enhance medication adherence and help sustain long‐term therapeutic outcomes, with potential additional benefits including relapse prevention, better tolerability, and reduced mortality (Ostuzzi et al. 2022; Johnson 2009). However, clinicians' reluctance to prescribe LAIAs is often driven by concerns regarding side effects (SEs) and the misconception that LAIAs carry a greater SE burden than OAPs (Correll et al. 2016), although evidence does not consistently support this; meta‐analyses indicate that oral and depot forms of the same antipsychotic drug do not differ significantly in extrapyramidal side effects or the likelihood of developing tardive dyskinesia (Adams et al. 2001; Omi et al. 2017), and, due to their pharmacokinetic properties, LAIAs may produce fewer dose‐related SEs than OAPs at comparable doses (Leucht et al. 2011).

Paliperidone palmitate is available in one‐month (PP1M) and 3‐month (PP3M) formulations. While the efficacy and tolerability of PP1M are well documented (Fernández‐Miranda et al. 2021; Katz et al. 2016; Pai and Warden 2018; Devrimci‐Özguven et al. 2019), data on the safety profile of PP3M—especially in real‐world clinical settings and at varying doses—remain limited (McEvoy et al. 2014; Naber et al. 2015; Pandina et al. 2011; Brasso et al. 2017; Fernández‐Miranda et al. 2021; Katz et al. 2016; Pai and Warden 2018). The clinical utility of PP1M has also been supported by national real‐world studies. For instance, Devrimci‐Özguven et al. (2019) conducted a multicenter mirror‐image study in Turkey, demonstrating significant reductions in both symptom severity and hospitalization rates following PP1M initiation. Most comparative studies have focused on different LAIAs rather than the transition from PP1M to PP3M. Furthermore, many existing studies have been industry‐sponsored, raising concerns about potential reporting bias (Brasso et al. 2017; Fleischhacker et al. 2012).

PP3M is currently the only LAIA with a 3‐month dosing interval, offering improved treatment convenience. Independent real‐world within‐patient data comparing PP1M and PP3M tolerability remain limited. Given the scarcity of independent, non‐sponsored studies directly examining the safety and tolerability of transitioning from PP1M to PP3M, further investigation is warranted. Therefore, present study aimed to evaluate within‐patient changes in side‐effect burden after conversion from PP1M to PP3M; we hypothesized that UKU scores would not increase after conversion. Specifically, the study sought to determine whether the clinical benefits and tolerability observed with PP1M were maintained or altered following the switch to PP3M.

## 2 Materials and Methods

This study utilized clinical data from 473 patients diagnosed with schizophrenia who presented to the outpatient clinic, Community Mental Health Center, and inpatient unit over a three‐month period between September 2021 and December 2021. This study was designed as a non‐interventional, observational, and naturalistic follow‐up of patients with schizophrenia who were initiated on LAI treatment as part of routine clinical care. All treatment decisions, including dose adjustments and adjunctive medication use, were made by the treating psychiatrists in the context of routine care without interference from the study team. A total of 132 out of 473 patients were initiated on LAIAs during the study period based on structured clinical criteria. In line with the *American Psychiatric Association Practice Guideline for the Treatment of Patients With Schizophrenia* (3rd edition, 2020), patients selected for LAIA treatment in this study met one or more of the following criteria: documented history of nonadherence to OAPs, repeated relapses or prior hospitalizations, poor insight or engagement with outpatient care, and/or a preference for long‐acting formulations. The LAIAs administered included paliperidone palmitate, risperidone long‐acting injection, aripiprazole monohydrate, haloperidol decanoate, flupentixol decanoate, fluphenazine decanoate, and zuclopenthixol decanoate. In the present study, 67 patients who had been switched to PP1M and met the inclusion criteria were enrolled. Among these, data from 43 patients who had received a stable dose of PP1M for at least 4 months before being transitioned to PP3M were included in the final analysis. Inclusion criteria were: (a) a diagnosis of schizophrenia according to the DSM‐5 diagnostic criteria; (b) age between 18 and 65 years; (c) provision of written informed consent by the patient or a legal representative; and (d) switching to PP1M, as deemed appropriate by the treating psychiatrist based on clinical evaluation. Exclusion criteria were as follows: (a) patients not meeting the criteria for switching to PP3M conversion, inclusion for PP3M conversion: (i) ≥ 4 months of PP1M at a stable dose; (ii) clinical stability and perceived benefit. Exclusion: failure to meet either (i) or (ii); (b) any modification to antipsychotic treatment not attributable to side effects, including: 1. Addition of an OAP to the regimen after the first PP1M injection, 2. Switching to another antipsychotic agent; (c) presence of an ongoing neurodevelopmental disorder or intellectual disability; (d) diagnosis of alcohol or substance use disorder; (e) treatment interruption, defined as a delay of more than seven consecutive days from the scheduled injection date; and (f) loss to follow‐up during the study period.

Of the 67 patients initially enrolled, 24 were excluded from the analysis: 13 due to unstable dosing or treatment changes following the first dose of PP1M (dose increase in 5 patients, dose decrease in 6 patients, and addition of OAPs in 2 patients); 6 due to dropout; and 5 due to nonadherence, defined as interruption of treatment for more than 1 week. The final analysis included 43 patients.

Baseline assessments were conducted during a period of symptomatic exacerbation. Patients had either recently discontinued their treatment, experienced clinical worsening due to nonadherence to OAPs, or presented with a first episode of psychosis accompanied by poor insight. Baseline evaluations included the administration of the Sociodemographic Information Form, along with the SANS and SAPS to assess the extent of negative and positive symptomatology. Patients who were initiated on PP1M and maintained a stable dose for 4 months were subsequently switched to PP3M. These participants were reassessed using the SANS, SAPS, and the UKU Side Effect Rating Scale at two follow‐up time points: 4 months after initiating PP1M and 4 months after switching to PP3M. No study‐specific interventions were implemented. Data were derived from routine clinical care documentation, complemented by scheduled research ratings performed at predefined time points.

Patients were stratified based on their antipsychotic regimen—monotherapy or polypharmacy—in order to control for potential confounding effects when evaluating side effects and functional outcomes. In this study, polypharmacy was defined as the concurrent use of paliperidone palmitate (PP1M or PP3M) with at least one additional psychotropic agent—such as another antipsychotic, an antidepressant, or a mood stabilizer—for a minimum duration of four consecutive weeks. Patients receiving PP monotherapy formed one group, while those receiving PP in combination with another psychotropic agent formed the *polypharmacy* group. Importantly, no change in the identity or dosage of the concomitant medication (e.g., clozapine, antidepressants) occurred between the PP1M and PP3M assessment phases. This ensured that any observed difference in side effects could not be attributed to changes in co‐medication regimens. PP3M doses were categorized as “lower” (175 mg or 263 mg) and “higher” (350 mg or 525 mg), based on the four available dose formulations of PP3M.

The research protocol received approval from the relevant ethics board (Approval ID: 90139838‐000‐E.20099). The study was conducted following the 2013 revision of the Declaration of Helsinki. Written consent forms were signed voluntarily by each participant before any data were collected.

### 2.1 Measurement Tools and Assessment Forms

#### 2.1.1 Sociodemographic Information Form

This form was specifically designed to gather detailed information regarding participants' demographic profiles and clinical history.

#### 2.1.2 Scale for the Assessment of Positive Symptoms

This scale consists of 34 items, each evaluated on a six‐point Likert scale, and is used to determine the presence, distribution, and intensity of positive symptoms in individuals diagnosed with schizophrenia (Andreasen and Olsen 1982). The validity of the Turkish version was established by Erkoç, Arkonaç, Ataklı, and Özmen (1991a).

#### 2.1.3 Scale for the Assessment of Negative Symptoms

Comprising 25 items and also scored using a six‐point scale, this instrument evaluates the frequency and severity of negative symptomatology (Andreasen and Olsen 1982). The Turkish version was similarly adapted and validated by Erkoç, Arkonaç, Ataklı, and Özmen (1991b).

#### 2.1.4 UKU Side Effect Rating Scale

This comprehensive 48‐item tool is used to evaluate the side effects associated with antipsychotic drug use. It includes four distinct subdomains: psychic, neurological, autonomic, and miscellaneous effects (Lingjaerde et al. 1987).

### 2.2 Statistical Analysis

All statistical analyses were conducted using IBM SPSS Statistics for Windows, version 26 (IBM Corp., Armonk, NY, USA). The normality of continuous data was examined based on skewness and kurtosis values, with acceptable limits defined between −2 and +2 (George and Mallery 2003). Descriptive statistics were reported as mean and standard deviation (SD) for normally distributed continuous variables, median and interquartile range (IQR) for non‐normally distributed continuous variables, and frequency (*n*) and percentage (%) for categorical variables. Categorical variables were compared using the chi‐square test; in cases where chi‐square assumptions regarding expected cell counts were violated, Fisher's exact test (for 2 × 2 tables) or Fisher–Freeman–Halton test (for larger tables) was used. For repeated measures, Repeated Measures ANOVA was applied to normally distributed data. When significant effects were found, pairwise comparisons were conducted using paired‐samples *t*‐tests with Bonferroni correction (by multiplying each *p*‐value by the number of comparisons). The Wilcoxon Signed‐Rank test was employed to compare pre‐ and post‐treatment symptom scores and side effects, while the Friedman test was used to evaluate differences across multiple time points. Effect sizes for ANOVA were calculated using partial eta squared (ηp^2^), with thresholds defined as small (ηp^2^ = 0.01), medium (0.06), and large (≥ 0.14) (Pallant 2020). For the Friedman test, Kendall's W coefficient was used to assess effect size, interpreted as small (*W* = 0.10), medium (0.30), and large (≥ 0.50) (Pallant 2020).^29^ Pearson's Chi‐Square test was applied to assess differences between categorical variables, while McNemar's test was utilized for evaluating categorical changes across time points. Effect size for McNemar's test was calculated using phi (*φ*), where *φ* = 0.10 indicates a small, 0.30 a medium, and ≥ 0.50 a large effect. A *p*‐value < 0.05 was considered statistically significant.

## 3 Results

The mean age of the 43 participants who completed the study was 35.16 ± 11.37 years, and the mean duration of education was 12.53 ± 2.94 years. Among the participants, 44.2% (*n* = 19) were female, 67.4% (*n* = 29) were single, 53.5% (*n* = 23) were unemployed, and 27.9% (*n* = 12) lacked family support. A history of homicidal ideation was reported by 9.3% (*n* = 4), and suicidal ideation by 27.9% (*n* = 12). Poor self‐care was observed in 39.5% (*n* = 17) of participants.

The mean duration of illness was 8.93 ± 8.85 years, with an average of 2.21 ± 4.13 hospitalizations and 4.14 ± 3.26 psychotic episodes. The mean number of attempts to discontinue oral medication was 3.18 ± 2.72.

When the patients' data after the fourth dose of PP1M were compared to baseline data, improvements were observed in the SANS and SAPS scores. The improvements in SANS scores remained stable after switching to PP3M, while the reduction in SAPS scores continued after the switch. Large effect sizes were observed in all SANS and SAPS subscales, except for delusions, which demonstrated a medium effect size (Table 1, Table 2).

**TABLE 1:** SANS scores of the participants at baseline, after PP1M, and after PP3M.

SANS subscale	Baseline mean ± SD	PP1M mean ± SD	PP3M mean ± SD	p* (PP1M vs baseline)	p* (PP3M vs baseline)	p* (PP3M vs PP1M)	ηp^2^
Avolution	14.20 ± 5.11	5.97 ± 3.90	5.45 ± 3.22	< 0.001	< 0.001	0.213	0.77
Attention	11.88 ± 3.53	4.95 ± 1.91	4.72 ± 2.62	< 0.001	< 0.001	1.000	0.83
Total score	73.79 ± 26.13	31.18 ± 14.28	28.25 ± 16.25	< 0.001	< 0.001	0.279	0.81

**SANS subscale**	**Baseline median (IQR)**	**PP1M median (IQR)**	**PP3M median (IQR)**	p** (PP1M vs baseline)	p** (PP3M vs baseline)	p** (PP3M vs PP1M)	**Kendall's W**
Affective flattening/blunting	23 (10)	10 (9)	7 (7)	< 0.001	< 0.001	0.453	0.81
Alogia	9 (11)	4 (8)	4 (6)	< 0.001	< 0.001	1.000	0.55
Anhedonia	20 (6)	7 (6)	5 (6)	< 0.001	< 0.001	1.000	0.82

Abbreviations: Kendall's W, kendall's coefficient of concordance; IQR, interquantile range; SANS, scale for the assessment of negative symptoms; SD, standard deviation; ηp^2^, partial eta squared; p*, adjusted *p* value obtained from paired‐samples *t* tests with bonferroni correction; p**, bonferroni adjusted *p*‐values obtained from pairwise comparisons using the wilcoxon signed rank test.

**TABLE 2:** SAPS scores of the participants at baseline, after PP1M, and after PP3M.

SAPS subscale	Baseline mean ± SD	PP1M mean ± SD	PP3M mean ± SD	p* (PP1M vs baseline)	p* (PP3M vs baseline)	p* (PP3M vs PP1M)	ηp^2^
Bizarre behavior	13.39 ± 5.72	3.41 ± 2.76	2.62 ± 2.29	< 0.001	< 0.001	0.687	0.80
Positive formal thought disorder	30.37 ± 11.50	6.46 ± 3.86	4.76 ± 2.60	< 0.001	< 0.001	< 0.001	0.84
Inappropriate affect	3.76 ± 0.75	1.41 ± 0.79	1.23 ± 0.68	< 0.001	< 0.001	0.309	0.87
Total score	73.72 ± 21.43	18.58 ± 8.88	14.06 ± 6.86	< 0.001	< 0.001	< 0.001	0.89

**SAPS subscale**	**Baseline median (IQR)**	**PP1M median (IQR)**	**PP3M median (IQR)**	p** (PP1M vs baseline)	p** (PP3M vs baseline)	p** (PP3M vs PP1M)	**Kendall's W**
Hallucinations	0 (9)	0 (0)	0 (0)	0.240	0.096	1.000	0.87
Delusions	22 (11)	6 (5)	5 (5)	< 0.001	< 0.001	0.045	0.30

Abbreviations: Kendall's W, kendall's coefficient of concordance; IQR, interquantile range; SAPS, scale for the assessment of positive symptoms; SD, standard deviation; ηp^2^, partial eta squared; p*, adjusted *p* value obtained from paired‐samples *t* tests with bonferroni correction; p**, bonferroni adjusted *p*‐values obtained from pairwise comparisons using the wilcoxon signed rank test.

Evaluation of UKU side effect data in patients receiving monotherapy revealed that the most frequently reported SEs following both PP1M and PP3M administration were increased fatigability (*n* = 14 [56%] and *n* = 13 [52%], respectively) and concentration difficulties (*n* = 14 [56%] for both) in the psychic subdomain; hypokinesia (*n* = 10 [40%] and *n* = 8 [32%], respectively) and akathisia (*n* = 6 [24%] and *n* = 5 [20%], respectively) in the neurological subdomain; and weight gain (*n* = 8 [32%] and *n* = 6 [24%], respectively) and reduced sexual desire (*n* = 7 [28%] for both) in the other subdomain. Most of these side effects were assessed as being of mild to moderate severity. The incidence of constipation significantly declined following the switch from PP1M to PP3M, and a medium‐to‐large effect size was observed (Table 3).

**TABLE 3:** Side effects after PP1M and PP3M.

	Side effects after PP1M				Side effects after PP3M				*φ*	p*
	NO *n* (%)	YES			NO *n* (%)	YES				
		mild *n* (%)	modarete *n* (%)	severe *n* (%)		mild *n* (%)	modarete *n* (%)	severe *n* (%)		
Side effects										
Psychic side effects										
Increased fatigability	14 (32.6)	18 (41.9)	11 (25.6)	—	17 (39.5)	22 (51.2)	4 (9.3)	—	0.18	0.250*
Concentration difficulties	15 (34.9)	15 (34.9)	12 (27.9)	1 (2.3)	16 (37.2)	23 (53.5)	4 (9.3)	—	0.00	1.000*
Sedation	16 (37.2)	16 (37.2)	11 (25.6)	—	21 (48.8)	18 (41.9)	4 (9.3)	—	0.27	0.063*
Emotional indifference	20 (46.5)	11 (25.6)	12 (27.9)	—	25 (58.1)	15 (34.9)	2 (4.7)	1 (2.3)	0.27	0.063*
Increased duration of sleep	21 (48.8)	12 (27.9)	10 (23.3)	—	25 (58.1)	14 (32.6)	3 (7.0)	1 (2.3)	0.19	0.219*
Failing memory	21 (48.8)	13 (30.2)	9 (20.9)	—	24 (55.8)	16 (37.2)	3 (7.0)	—	0.17	0.250*
Depression	22 (51.2)	12 (27.9)	9 (20.9)	—	25 (58.1)	15 (34.9)	3 (7.0)	—	0.17	0.250*
Neurological side effects										
Hypokinesia	23 (53.5)	15 (34.9)	5 (11.6)	—	29 (67.4)	12 (27.9)	1 (2.3)	1 (2.3)	0.27	0.070*
Akathisia	31 (72.1)	9 (20.9)	3 (7.0)	—	32 (74.4)	10 (23.3)	—	1 (2.3)	0.00	1.000*
Tremor	34 (79.1)	8 (18.6)	1 (2.3)	—	34 (79.1)	8 (18.6)	1 (2.3)	—	0.00	1.000*
Dystonia	36 (83.7)	2 (4.7)	5 (11.6)	—	40 (93.0)	2 (4.7)	—	1 (2.3)	0.23	0.125*
Rigidity	37 (86.0)	4 (9.3)	2 (4.7)	—	42 (97.7)	—	—	1 (2.3)	0.27	0.063*
Headache	37 (86.0)	5 (11.6)	1 (2.3)	—	38 (88.4)	4 (9.3)	1 (2.3)	—	0.00	1.000*
Autonomic side effects										
Constipation	26 (60.5)	11 (25.6)	6 (14.0)	—	33 (76.7)	7 (16.3)	3 (7.0)	—	**0.35**	0.016*
Other side effects										
Weight gain	23 (53.5)	10 (23.3)	10 (23.3)	—	26 (60.5)	10 (23.3)	6 (14.0)	1 (2.3)	0.17	0.250*
Diminished sexual desire	25 (58.1)	8 (18.6)	9 (20.9)	1 (2.3)	27 (62.8)	14 (32.6)	1 (2.3)	1 (2.3)	0.11	0.500*
Erectile dysfunction	33 (76.7)	8 (18.6)	2 (4.7)	—	35 (81.4)	8 (18.6)	—	—	0.11	0.500*
Amenorrhea	34 (79.1)	4 (9.3)	5 (11.6)	—	34 (79.1)	8 (18.6)	1 (2.3)	—	0.00	1.000*
Ejaculatory dysfunction	36 (83.7)	6 (14.0)	—	1 (2.3)	38 (88.4)	4 (9.3)	1 (2.3)	—	0.11	0.500*

*Note:* Bold values denote statistically significant results (*p* < 0.05).

Abbreviations: *n*, number of cases; p*, mcnemar chi square test statistical significance value; *φ*, the effect size for the mcnemar test.

When side effects were compared across antipsychotic treatment groups, weight gain and reduced sexual desire were found to be more prevalent among patients receiving polypharmacy. Among less common adverse effects, erectile (*p* = 0.009) and ejaculatory dysfunctions (*p* = 0.015) were also more frequent in this group. In males (*n* = 24), the polypharmacy subgroup comprised 15 men and the monotherapy subgroup comprised 9 men. Erectile and ejaculatory dysfunctions were more frequent in the polypharmacy group (erectile dysfunction: 7/15 vs 1/9; ejaculatory dysfunction: 5/15 vs 0/9). Additionally, weight gain was significantly more frequent among patients using multiple antipsychotics after PP3M as well. Increased salivation was significantly more common in the polypharmacy group (*p* = 0.034) (Table 4).

**TABLE 4:** Comparison of common side effects according to PP1M/PP3M dose and treatment classification.

	Side effect		Treatment classification			PP1M dose					PP1M dose		
			Monotherapy	Polifarmacy	p*	50 mg	75 mg	100 mg	150 mg	p**	< 100 mg	≥ 100 mg	p*
UKU (after PP1M)	Increased fatigability	Yes	14	15	0.059	6	10	6	7	0.204	16	13	0.750
		No	11	3		6	1	3	4		7	7
	Concentration difficulties	Yes	14	14	0.139	6	10	6	6	0.172	16	12	0.512
		No	11	4		6	1	3	5		7	8	
	Sedation	Yes	14	13	0.278	6	9	6	6	0.417	15	12	0.724
		No	11	5		6	2	3	5		8	8	
	Emotional indifference	Yes	13	10	0.818	7	8	4	4	0.349	15	8	0.098
		No	12	8		5	3	5	7		8	12	
	Increased duration of sleep	Yes	11	11	0.268	6	8	3	5	0.379	14	8	0.172
		No	14	7		6	3	6	6		9	12	
	Failing memory	Yes	13	9	0.897	6	9	4	3	0.085	15	7	**0.048**
		No	12	9		6	2	5	8		8	13	
	Depression	Yes	10	11	0.172	4	8	4	5	0.319	12	9	0.639
		No	15	7		8	3	5	6		11	11	
	Weight gain	Yes	8	12	**0.025**	4	8	3	5	0.231	12	8	0.425
		No	17	6		8	3	6	6		11	12	
	Hypokinesia	Yes	10	10	0.313	5	4	6	5	0.631	9	11	0.298
		No	15	8		7	7	3	6		14	9	
	Diminished sexual desire	Yes	7	11	**0.030**	5	6	3	4	0.827	11	7	0.395
		No	18	7		7	5	6	7		12	13	

			**Treatment classification**			**PP3M dose**					**PP3M dose**		
			**Monotherapy**	**Polifarmacy**	p*	**175 mg**	**263 mg**	**350 mg**	**520 mg**	p**	**< 350 mg**	**≥ 350 mg**	p*
UKU (after PP3M)	Concentration difficulties	Yes	14	13	0.278	7	8	6	6	0.585	15	12	0.724
		No	11	5		6	2	3	5		8	8	
	Increased fatigability	Yes	13	13	0.181	5	9	6	6	0.085	14	12	0.954
		No	12	5		8	1	3	5		9	8	
	Sedation	Yes	13	9	0.897	6	7	4	5	0.620	13	9	0.451
		No	12	9		7	3	5	6		10	11	
	Failing memory	Yes	12	7	0.553	5	7	4	3	0.266	12	7	0.258
		No	13	11		8	3	5	8		11	13	
	Increased duration of sleep	Yes	9	9	0.359	5	6	2	5	0.471	11	7	0.395
		No	16	9		8	4	7	6		12	13	
	Emotional indifference	Yes	11	7	0.738	5	6	3	4	0.647	11	7	0.395
		No	14	11		8	4	6	7		12	13	
	Depression	Yes	10	8	0.771	4	6	3	5	0.524	10	8	0.816
		No	15	10		9	4	6	6		13	12	
	Weight gain	Yes	6	11	**0.014**	4	5	3	5	0.786	9	8	0.954
		No	19	7		9	5	6	6		14	12	
	Diminished sexual desire	Yes	7	9	0.141	5	4	3	4	1.000	9	7	0.780
		No	18	9		8	6	6	7		14	13	
	Hypokinesia	Yes	8	6	0.927	3	4	3	4	0.847	7	7	0.750
		No	17	12		10	6	6	7		16	13	

*Note:* Bold values denote statistically significant results (*p* < 0.05).

Abbreviations: PP1M, paliperidone palmitate‐one‐month; PP3M, paliperidone palmitate‐three‐month; p*, chi‐square test statistical significance value; p**, fisher‐freeman‐halton test statistical significance value; UKU, UKU side effect rating scale.

Regarding the association between dosage and side effects, our findings indicate that increasing the dose was not associated with a significant rise in overall side effect frequency (Table 4). However, amenorrhea (*p* = 0.024) was more frequently observed in the high‐dose group, whereas failing memory was significantly less common.

Among those treated solely with PP1M, 40% reported mild and 24% moderate side effects. In this group, 16% discontinued medication due to side effects, and 16% required additional treatment. In the PP3M‐only group, 48% reported mild, 8% moderate, and 8% severe side effects. Within this group, 12% discontinued medication due to side effects, 8% required additional treatment, and medication was completely withdrawn in 8% (Table 5). Two patients in our study discontinued treatment due to severe SEs. One 63‐year‐old patient experienced rigidity that began approximately 4 weeks after the second 175 mg PP3M dose, following four consecutive PP1M injections at 50 mg. The patient was treated with biperiden 4 mg/day, with gradual week‐by‐week improvement, and symptoms fully resolved by the end of the second month. The second patient developed severe akathisia beginning approximately 3 weeks after a 263 mg PP3M dose. Propranolol 80 mg/day was initially trialed without benefit; diazepam 10 mg/day was subsequently initiated, leading to partial symptom reduction, with complete resolution occurring over several months (akathisia had resolved by month 4).

**TABLE 5:** Frequency of global side effects and consequences at the end of the fourth month of patients using PP1M‐PP3M.

Monotherapy					Polypharmacy				
Global side effects	NO *n* (%)	YES *n* (%)			Global side effects	NO *n* (%)	YES *n* (%)		
		Mild	Moderate	Severe			Mild	Moderate	Severe
After PP1M	9 (36)	10 (40)	6 (24)	—	After PP1M	1 (5)	9 (50)	8 (44)	—
After PP3M	9 (36)	12 (48)	2 (8)	2 (8)	After PP3M	5 (27)	10 (55)	3 (16)	—

**Consequence**	NO ACTION *n* (%)	YES *n* (%)			**Consequence**	NO ACTION *n* (%)	YES *n* (%)		
		More frequent assessment of the patient. But no reduction of dose, and/or occasional drug treatment of side effects	Reduction of dose and/or continuous drug treatment of side effects	Discontinuation of drug or change to another preparation			More frequent assessment of the patient. But no reduction of dose, and/or occasional drug treatment of side effects	Reduction of dose and/or continuous drug treatment of side effects	Discontinuation of drug or change to another preparation
After PP1M	17 (68)	4 (16)	4 (16)	—	After PP1M	8 (44)	4 (22)	6 (33)	—
After PP3M	18 (72)	3 (12)	2 (8)	2 (8)	After PP3M	11 (61)	6 (33)	1 (5)	—

Abbreviations: *n*, number of cases; PP1M, paliperidon palmitate‐one‐month; PP3M, paliperidon palmitate‐three‐month.

## 4 Discussion

The present study systematically evaluated adverse events in a real‐world, naturalistic setting using an observational design to compare the monthly and three‐monthly formulations of paliperidone palmitate. Treatment decisions, including the initiation and continuation of LAIAs or the addition of other antipsychotics, were made by the attending psychiatrists as part of standard clinical practice. Our primary finding is that the switch from PP1M to PP3M was not associated with an increase in overall side‐effect burden as assessed by the UKU Side Effect Rating Scale. The most frequently reported adverse effects—including increased fatigability, hypokinesia, weight gain, and reduced sexual desire—remained stable after the switch, and no new safety signals emerged. These findings indicate that extending the dosing interval to 3 months does not adversely affect tolerability in patients stabilized on PP1M.

Our findings are consistent with the pivotal randomized, double‐blind, phase‐3 noninferiority study by Savitz et al. (2016), which demonstrated comparable safety and tolerability profiles for PP3M and PP1M over a 48‐week double‐blind phase. In that study, weight gain was the most common treatment‐emergent adverse event in both groups, while rates of extrapyramidal symptoms, QT prolongation, prolactin‐related effects, and treatment discontinuation due to adverse events were similar. Similarly, the subgroup analysis by Savitz et al. (2019) confirmed comparable tolerability between PP3M and PP1M across European and non‐European populations, despite higher overall adverse‐event rates in non‐European—particularly Asian—patients. However, several methodological differences between our study and the Savitz trials merit consideration. The Savitz studies were conducted using randomized, double‐blind designs with prolonged open‐label stabilization phases, strict clinical stability criteria, oral tolerability testing, fixed dosing strategies, and placebo injections to maintain blinding, thereby enrolling a highly selected and stabilized patient population. In contrast, our study reflects routine clinical practice, without protocol‐driven stabilization phases or restrictive exclusion of patients receiving concomitant psychotropic medications. These design differences underscore the complementary nature of our findings, extending evidence from controlled clinical trials to a more heterogeneous real‐world patient population.Consistent with the safety findings reported in the Savitz trials, weight gain emerged as the most frequently reported adverse effect in our cohort, particularly within the “other” subscale of the UKU scale. This finding aligns with previous clinical trials identifying metabolic adverse effects as common with paliperidone palmitate (Hough et al. 2010; Nasrallah et al. 2010). Notably, Devrimci‐Özguven et al. (2019) also identified weight gain as a prevalent adverse event in their multicenter real‐world cohort treated with PP1M. Importantly, despite the more heterogeneous and less selectively stabilized patient population in our naturalistic sample, no statistically significant difference in weight gain was observed between PP1M and PP3M. In line with earlier reports, increases in body mass index appeared to follow a dose‐related pattern rather than being driven by dosing interval, particularly among patients receiving higher PP3M doses (Nasrallah et al. 2010). Taken together, these findings suggest that metabolic adverse effects associated with paliperidone palmitate are predictable and clinically manageable with appropriate monitoring, even in real‐world treatment settings.

Neurological side effects, particularly hypokinesia, were also evaluated. While hypokinesia was more frequently observed in patients receiving PP1M (46.5%) compared to those switched to PP3M (33.4%), the difference was not statistically significant (*φ* = 0.27, *p* = 0.070). The within‐patient trend did not indicate a higher EPS burden after conversion; larger samples are needed to clarify any small differences. Consistent with prior reports, extrapyramidal symptoms with PP have typically been reported as mild and manageable (Hough et al. 2010).

Psychic SEs were also common, with increased mental fatigue being the most frequently reported symptom in the UKU psychic subscale. No significant difference was found between the two formulations in this regard (*φ* = 0.17, *p* = 0.250). Importantly, although some studies have suggested a potential link between PP and worsening of depressive symptoms (Gentile 2013), our results did not confirm this.

While the UKU scale offers a structured approach to side effect monitoring, it does not capture all potential SEs, such as injection site reactions or laboratory abnormalities (e.g., hyperprolactinemia, hyperlipidemia). Thus, our findings should be interpreted within this context. Nevertheless, our observation that 64% of patients experienced at least one SE with PP3M monotherapy is in line with a previous study, which reported a 58.5% incidence rate (Najarian et al. 2022).

One of the most clinically relevant findings of this study is that increased PP doses were not associated with a higher incidence of SEs. This suggests that, following appropriate titration and stabilization, PP3M can be used with acceptable tolerability across dose tiers. Notably, tolerability remained acceptable even at the upper end of the approved range, including the highest available strength (525 mg), as supported by our dose‐tier analyses and consistent with previous studies (Fernández‐Miranda et al. 2021). Additionally, our data supports the strategy of monotherapy over polypharmacy. Patients on PP monotherapy had fewer SEs, particularly regarding weight gain and reduced sexual desire, compared to those receiving PP in combination with other APs. These findings align with studies reporting a higher burden of SEs—including sleep disturbance, dystonia, and sexual dysfunctions—in patients on multiple Aps (Ceylan et al. 2016).

In terms of SE management, 68% of patients in the PP1M monotherapy group and 72% in the PP3M monotherapy group required no clinical intervention. Only 8% of patients in the PP3M group discontinued treatment due to SEs. These results suggest that, with appropriate patient selection and monitoring, PP3M is a well‐tolerated and clinically viable long‐term treatment option for schizophrenia.

Although clinicians may be concerned that the long half‐life of PP3M could complicate the management of emerging SEs (Bioque and Bernardo 2018), our findings do not support this concern. On the contrary, switching from PP1M to PP3M did not result in a significant increase in SE burden and may even reduce certain symptoms in specific cases. Furthermore, data from UKU evaluations indicate that the overall burden of SEs may be lower in PP‐treated patients compared to those on second‐generation OAPs (Aykut 2019).

In schizophrenia treatment, LAIAs offer several clinical advantages, including improved medication adherence, consistent plasma drug levels, and reduced relapse and hospitalization rates (Verdoux et al. 2017; Peters et al. 2019; Rise et al. 2021; Taipale et al. 2018). PP3M, as the only available 3‐month LAIA, further enhances convenience by extending the injection interval, which may improve adherence and reduce stigma related to frequent treatment (Pietrini et al. 2019; Najarian et al. 2022).

In line with previous studies, our findings indicate significant reductions in symptom severity following the initiation of PP1M, particularly in positive symptoms, as reflected by the improvement in SAPS scores with a large effect size (ηp^2^ = 0.89, *p* < 0.001). Similarly, significant improvements in negative symptoms were observed following treatment (ηp^2^ = 0.81, *p* < 0.001). These results are consistent with prior real‐world evidence. Supporting this, Devrimci‐Özguven et al. (2019) reported robust clinical gains and enhanced treatment adherence in a multicenter Turkish cohort. These findings align with large‐scale studies showing that both PP1M and PP3M lead to meaningful symptom improvements and that switching to PP3M maintains clinical effectiveness (Gopal et al. 2020; Savitz et al. 2016). However, it should be noted that our primary focus was not efficacy per se, but rather the tolerability and SE profile associated with the treatment transition.

This study has several important limitations. Primarily, the relatively small sample size and its confinement to a single clinical site may limit the broader applicability of the results. Second, as the study employed an observational design, it is not possible to draw definitive causal inferences between medication exposure and the side effects observed. Third, side effects were assessed using the UKU Side Effect Rating Scale, which, although comprehensive, does not include open‐ended questions or capture all possible adverse events, such as injection site reactions or laboratory abnormalities like hyperprolactinemia or dyslipidemia. Fourth, biochemical parameters and prolactin levels were not systematically monitored, which would have provided additional insight into metabolic and hormonal side effects. Fifth, the exclusion of patients with unstable dosing or those on changing antipsychotic regimens may have resulted in selection bias by focusing on a relatively stable patient population. Finally, the follow‐up period after switching to PP3M was limited to 4 months, which may not have been sufficient to capture late‐onset side effects or long‐term tolerability outcomes. Moreover, longer follow‐up after initiating a long‐acting injectable antipsychotic (e.g., ≥ 6 months) is often recommended in clinical practice to appraise maintenance outcomes and tolerability; accordingly, our 4‐month post‐switch follow‐up may miss late‐onset adverse events and limits inference regarding longer‐term outcomes. Also, the relatively small sample size and single‐center design restrict the generalizability of our findings. Longer follow‐up periods with larger, multicenter cohorts are warranted to validate and expand upon our findings. Despite these limitations, the study adds valuable real‐world data on the tolerability of PP3M, especially in comparison to PP1M, and provides insight into the clinical management of antipsychotic treatment in routine practice.

## 5 Conclusions

The findings of this within‐patient comparison (PP1M month‐4 to PP3M month‐4) indicate that conversion to PP3M was not associated with an increase in overall UKU side‐effect burden. In patients who were appropriately dosed before switching, symptom control appeared to be maintained, and adverse events did not significantly differ in frequency or severity after the transition. Findings highlight individualized dosing and a preference for monotherapy over antipsychotic polypharmacy to minimize adverse‐event load. The structured evaluation of side effects and the real‐world comparison of monotherapy versus polypharmacy use may offer useful insights into the clinical application of PP3M. Although further large‐scale and longer‐term studies are warranted to confirm and expand upon these findings, the present study contributes to the growing literature suggesting that, with careful patient selection and monitoring, PP3M could represent a feasible and acceptable option in the maintenance treatment of schizophrenia. These real‐world data can inform clinical decisions on LAIA continuation strategies after PP1M stabilization.

## Author Contributions

Concept: E.K., I.O., B.Y., M.H.T. Design: E.K., I.O., B.Y., M.H.T. Supervision: B.Y., M.H.T. Resources: E.K., I.O. Materials: E.K., I.O. Data Collection and/or Processing: E.K., I.O. Analysis and/or Interpretation: E.K., I.O., B.Y. Literature Search: E.K., I.O. Writing: E.K., I.O., B.Y. Critical Review: B.Y., M.H.T. All authors have read and agreed to the published version of the manuscript.

## Funding

All authors solemnly declare that no financial support has been received from any individual or company for the submitted work. This research did not receive any specific grant from funding agencies in the public, commercial, or not‐for‐profit sectors.

## Ethics Statement

Institutional Review Board Statement: The study was approved by the ethical committee of the University of Giresun, Turkey (Approval ID: 90139838‐000‐E.20099). This study was conducted in accordance with the Declaration of Helsinki.

## Consent

Written informed consent was obtained from all study participants.

## Conflicts of Interest

The authors declare no conflicts of interest.

## Data Availability

The data that support the findings of this study are available from the corresponding author upon reasonable request.
